# TIMAP inhibits endothelial myosin light chain phosphatase by competing with MYPT1 for the catalytic protein phosphatase 1 subunit PP1cβ

**DOI:** 10.1074/jbc.RA118.006075

**Published:** 2019-07-17

**Authors:** Xin Wang, Marya Obeidat, Laiji Li, Phuwadet Pasarj, Salah Aburahess, Charles F. B. Holmes, Barbara J. Ballermann

**Affiliations:** ‡ Department of Medicine, University of Alberta, Edmonton, Alberta T6G 2G3, Canada; § Department of Biochemistry, University of Alberta, Edmonton, Alberta T6G 2G3, Canada

**Keywords:** phosphoprotein phosphatase 1 (PP1), myosin, endothelial cell, inhibitor, angiogenesis, microcystin, myosin phosphatase, MYPT1

## Abstract

Transforming growth factor-β membrane associated protein (TIMAP) is an endothelial cell (EC)–predominant PP1 regulatory subunit and a member of the myosin phosphatase target (MYPT) protein family. The MYPTs preferentially bind the catalytic protein phosphatase 1 subunit PP1cβ, forming myosin phosphatase holoenzymes. We investigated whether TIMAP/PP1cβ could also function as a myosin phosphatase. Endogenous PP1cβ, myosin light chain 2 (MLC2), and myosin IIA heavy chain coimmunoprecipitated from EC lysates with endogenous TIMAP, and endogenous MLC2 colocalized with TIMAP in EC projections. Purified recombinant GST-TIMAP interacted directly with purified recombinant His-MLC2. However, TIMAP overexpression in EC enhanced MLC2 phosphorylation, an effect not observed with a TIMAP mutant that does not bind PP1cβ. Conversely, MLC2 phosphorylation was reduced in lung lysates from TIMAP-deficient mice and upon silencing of endogenous TIMAP expression in ECs. Ectopically expressed TIMAP slowed the rate of MLC2 dephosphorylation, an effect requiring TIMAP–PP1cβ interaction. The association of MYPT1 with PP1cβ was profoundly reduced in the presence of excess TIMAP, leading to proteasomal MYPT1 degradation. In the absence of TIMAP, MYPT1-associated PP1cβ readily bound immobilized microcystin-LR, an active-site inhibitor of PP1c. By contrast, TIMAP-associated PP1cβ did not interact with microcystin-LR, indicating that the active site of PP1cβ is blocked when it is bound to TIMAP. Thus, TIMAP inhibits myosin phosphatase activity in ECs by competing with MYPT1 for PP1cβ and blocking the PP1cβ active site.

## Introduction

Ser/Thr protein phosphatase 1 (PP1) holoenzymes are heteromers containing PP1 catalytic (PP1c) and regulatory subunits. The catalytic PP1c subunits PP1cα, PP1cβ (also known as PP1cδ), and PP1cγ and their splice variants are highly conserved, with PP1cα and PP1cγ more closely related to each other than to PP1cβ (1). The PP1c subunits associate with more than 200 diverse PP1c-interacting proteins (PPIPs)^6^ (2). Most PPIPs are holoenzyme regulatory subunits that define the substrate specificity, phosphatase activity, subcellular localization, and cell type specificity of the PP1 phosphatase they form with their PP1c partner (3). The PPIPs also include PP1c inhibitors (4–7) and PP1c substrates. Free PP1c subunits dephosphorylate protein Ser/Thr residues in an indiscriminate fashion, but in cells, PPIPs exist in molar excess and bind PP1c with high affinity, preventing nonspecific PP1c activity (2, 4, 6).

The myosin phosphatase target (MYPT) family of PPIPs consists of regulatory subunits of PP1 phosphatases that tightly control the rate of myosin light chain (MLC) dephosphorylation, regulating myosin activity. The N-terminal PP1c-binding domain of all MYPTs is structurally conserved, with an RAQQLKKW myosin phosphatase N-terminal element also known as the “MyPhoNE” motif (8), upstream of the consensus KVXF PP1c docking motif (9, 10), followed by several ankyrin repeats. This MYPT N-terminal domain reshapes the PP1c catalytic cleft to provide specificity for discrete substrates (11, 12). The MYPT C-terminal domain regulates PP1c activity. Notably, phosphorylation of MYPT1 by Rho-associated kinase (ROCK) at Thr^696^ and Thr^853^ inhibits the MYPT1/PP1cβ phosphatase activity toward MLC2, the regulatory light chain of nonmuscle myosin, increasing myosin activity (13–15).

The PPIPs TIMAP (PPP1R16B) and MYPT3 (PPP1R16A) form a subgroup in the MYPT family and share the N-terminal domain structure of the MYPTs (16). However, the C-terminal inhibitory phosphorylation sites found in MYPT1, MYPT2, and p85 are not conserved in TIMAP or MYPT3. Instead, TIMAP is phosphorylated by PKC on Ser^331^, blocking association with and dephosphorylating ERM (Ezrin Radixin Moesin) proteins (17). TIMAP is also phosphorylated on Ser^333^ and Ser^337^ by glycogen synthetase kinase β (GSK3β) and cAMP-dependent PKA, respectively, increasing *in vitro* phosphatase activity and slightly reducing the affinity of TIMAP for PP1cβ (18, 19). The MYPT3 C-terminal domain is similarly phosphorylated by PKA at Ser^353^, enhancing phosphatase activity (20). Finally, unlike other MYPTs, TIMAP and MYPT3 contain a CAA*X* motif at their extreme C terminus, resulting in prenylation and membrane association (21, 22).

TIMAP is most highly expressed in endothelial cells (ECs), leukocyte/megakaryocyte cell lines (22), and in the central nervous system (23). Functional effects of TIMAP have been described in ECs *in vivo* and *in vitro*. In this regard, the blood–tissue barrier function of the pulmonary capillary endothelium is at least partly myosin motor–dependent (24–26), and *in vivo*, pulmonary EC TIMAP enhances this barrier function (27, 28). Also, during angiogenesis, dynamic changes in EC position as tip or stalk cells and cellular elongation in angiogenic sprouts are myosin-dependent (29), and TIMAP is required for the formation of angiogenic sprouts by cultured ECs (30). Recombinant GST-TIMAP/PP1cβ can dephosphorylate MLC2 *in vitro* (11), suggesting that TIMAP/PP1cβ may be a myosin phosphatase holoenzyme. Nonetheless, a functional role for TIMAP/PP1cβ as a myosin phosphatase in cells has not been established.

We therefore sought to determine whether TIMAP/PP1cβ functions as a myosin phosphatase in living ECs. Contrary to expectations, we observed that TIMAP inhibits MLC2 dephosphorylation through competition with MYPT1 for PP1cβ. Furthermore, we found that the PP1cβ active site, although accessible when PP1cβ is bound to MYPT1, is blocked when PP1cβ is associated with TIMAP. The findings suggest that TIMAP functions as a PP1cβ inhibitor in living ECs.

## Results

### TIMAP colocalizes with MLC2 in cellular projections and can interact directly with MLC2 and pMLC2

**Figure 1.**
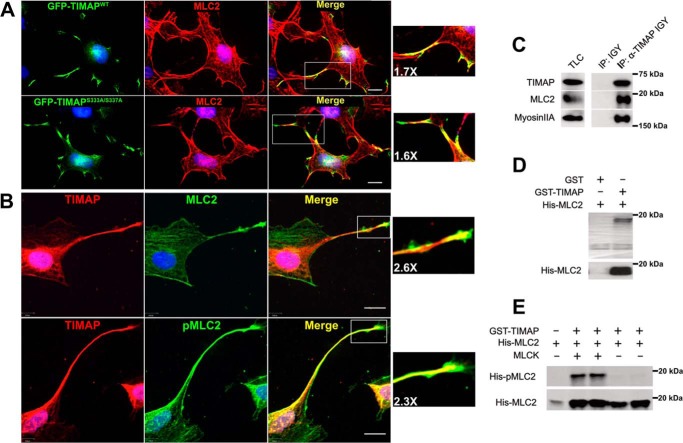
**TIMAP interacts directly with the myosin MLC2 subunit.** *A*, colocalization of overexpressed GFP-TIMAP^WT^ (*top panels*, *green*) and GFP-TIMAP^S333A/S337A^ (*bottom panels*, *green*) with MLC2 (*red*) in COS7 cells. *Scale bars* = 10 μm. Areas in *white boxes* were digitally magnified. *B*, immunoreactivity of anti-TIMAP (*red*), anti-MLC2 (*top panels*, *green*), and anti-pThr^18^/Ser^19^ MLC2 (*pMLC2*; *bottom panels*, *green*) antibodies with endogenous proteins in cultured glomerular ECs. *Scale bars* = 10 μm. Areas in *white boxes* were digitally magnified. *C*, endogenous TIMAP immunoprecipitated from glomerular EC lysates (*TLC*) with chicken anti-TIMAP IgY (*IP:* α*-TIMAP IGY*) or nonimmune IgY (*IP: IGY*), followed by WB analysis for TIMAP, MLC2, and Myosin IIA heavy chain. *D*, recombinant purified GST or GST-TIMAP immobilized on GSH beads was incubated with recombinant purified His-MLC2 *in vitro*, followed by precipitation of the beads. *Top*, Amido Black–stained blot. *Bottom*, anti-MLC2 WB analysis. *E*, recombinant His-MLC2 incubated *in vitro* in kinase buffer with or without MLCK, followed by pulldown with GST-TIMAP and WB analysis for pMLC2 and total MLC2.

We first determined whether TIMAP interacts with its putative substrate, MLC2 (Fig. 1). In COS7 cells, which do not express endogenous TIMAP, exogenously expressed GFP-TIMAP and GFP-TIMAP^S333A/S337A^, a mutant not phosphorylated by PKA/GSK-3β, were observed only in cellular projections, where they colocalized with MLC2 (Fig. 1*A*). Overexpression of GFP-TIMAP in ECs also colocalized in cellular projections with MLC2 (Fig. S1). Similarly, endogenous TIMAP was observed predominantly in EC projections, where it colocalized with MLC2 and pThr^18^/pSer^19^ MLC2 (pMLC2) (Fig. 1*B*), and endogenous MLC2 and the Myosin IIA heavy chain were coimmunoprecipitated with endogenous TIMAP from EC lysates (Fig. 1*C*). Because colocalization and coimmunoprecipitation cannot resolve whether proteins interact directly, we next determined whether TIMAP and MLC2 interact in the absence of other proteins. Recombinant purified His-MLC2 was pulled from solution *in vitro* by immobilized recombinant GST-TIMAP, indicating that His-MLC2 can interact directly with GST-TIMAP (Fig. 1*D*). This interaction was observed whether MLC2 was diphosphorylated by MLCK or not (Fig. 1*E*). Thus, endogenous TIMAP exists in a complex with myosin in EC projections, and TIMAP can interact directly with MLC2 and pMLC2.

### TIMAP expression leads to increased MLC2 phosphorylation

**Figure 2.**
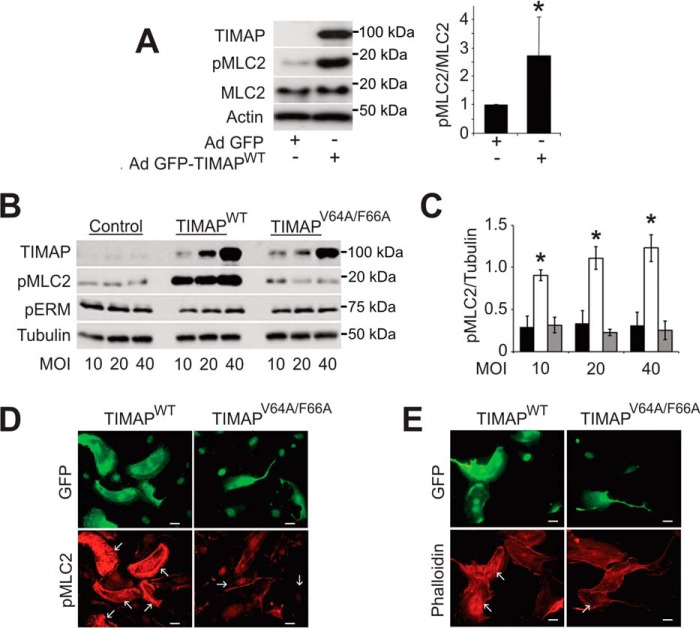
**TIMAP overexpression increases MLC2 phosphorylation and stress fiber formation in ECs.** *A*, GFP-TIMAP was overexpressed using an adenoviral (*Ad*) vector in glomerular ECs. *Left panel*, representative WB analysis. *Right panel*, quantitative analysis (mean ± S.D.; *n* = 3 independent experiments; *, *p* < 0.01). *B*, glomerular ECs were transduced with adenoviral GFP-TIMAP^WT^ or GFP-TIMAP^V64A/F66A^ at 10, 20, and 40 m.o.i., followed 48 h later by WB analysis for TIMAP, pMLC2, pERM, and tubulin. *C*, quantification of WB analysis from three independent experiments (*black columns*, GFP-vector; *white columns*, GFP-TIMAP^WT^; *gray columns*, GFP-TIMAP^V64A/F66A^; *, *p* < 0.001, two-way ANOVA with post hoc Bonferroni test for individual differences). *D*, glomerular ECs were transduced with adenoviral GFP-TIMAP^WT^ or GFP-TIMAP^V64A/F66A^ (m.o.i. 20). 48 h later, cells from each group were mixed 50:50 with glomerular ECs that had been transduced with GFP-vector. GFP-TIMAP^WT^ and GFP-TIMAP^V64A/F66A^ expression was observed throughout the cells, whereas GFP alone was nuclear in GFP-vector–transduced cells. pMLC2 immunofluorescence was markedly augmented in ECs expressing GFP-TIMAP^WT^ but not in ECs expressing GFP-TIMAP^V64A/F66A^. *E*, glomerular EC were transduced as in *D*, followed by evaluation for GFP fluorescence and phalloidin-labeled polymerized actin (*red*). Compared with vector-transduced cells, phalloidin labeling of stress fibers was enhanced in ECs expressing GFP-TIMAP^WT^ but not in ECs expressing GFP-TIMAP^V64A/F66A^. *Scale Bars* = 10 μm.

We next determined whether TIMAP expression alters MLC2 phosphorylation in ECs (Fig. 2). Contrary to previous findings that TIMAP/PP1cβ can dephosphorylate pMLC2 *in vitro*, MLC2 phosphorylation was much greater in ECs overexpressing GFP-WT TIMAP (GFP-TIMAP^WT^) compared with GFP-vector–transduced ECs (Fig. 2*A*). This effect of exogenously expressed TIMAP depended on the multiplicity of infection (m.o.i.) and was not observed with expression of GFP-TIMAP^V64A/F66A^, a TIMAP mutant that does not bind PP1c (18) (Fig. 2, *B* and *C*). Immunofluorescence microscopy similarly showed increased pMLC2 in ECs overexpressing GFP-TIMAP^WT^ but not in ECs overexpressing GFP-TIMAP^V64A/F66A^ (Fig. 2*D*). More stress fibers were observed in ECs transduced with GFP-TIMAP^WT^ than in ECs transduced with GFP-vector or GFP-TIMAP^V64A/F66A^ (Fig. 2*E*). His_6_-TIMAP^WT^ and GFP-TIMAP^WT^ similarly increased the pMLC2:MLC2 ratio in COS7 cells by 43.8% ± 0.19% and 34.2% ± 12%, respectively, compared with vector alone (mean ± S.D., *n* = 4 each, *p* = 0.01 and 0.005, respectively).

**Figure 3.**
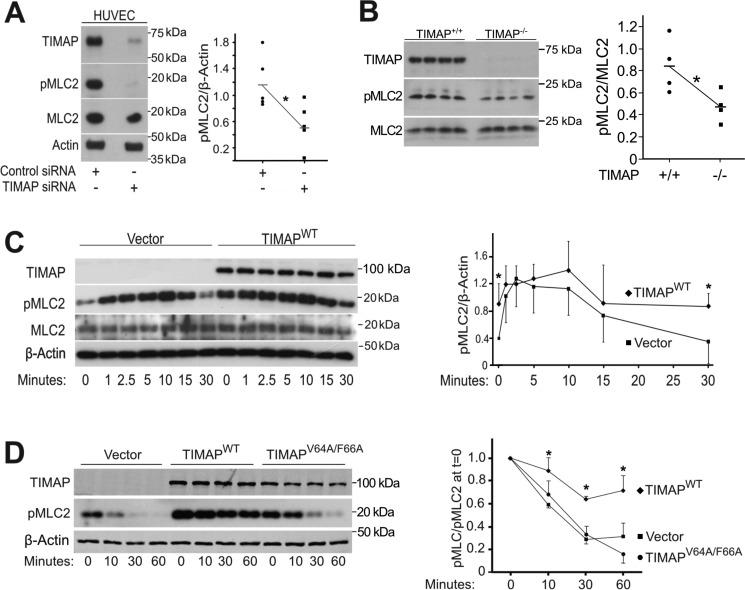
**TIMAP raises steady-state pMLC2 levels by reducing the rate of pMLC2 dephosphorylation.** *A*, cultured HUVECs were transfected with control or TIMAP-specific siRNA. *Left panel*, representative WB analysis. *Right panel*, densitometric analysis (*n* = 5 independent experiments; *, *p* < 0.01). Β, whole-lung lysates from WT (*TIMAP*^+/+^) and age-matched TIMAP-deficient (*TIMAP*^−/−^) mice. *Left panel*, WB analysis for TIMAP, pMLC2, and MLC2. Each lane represents lysate from a distinct mouse. *Right panel*, densitometric quantification of pMLC2/MLC2 (*n* = 4/group; *, *p* < 0.05). *C*, glomerular ECs transduced with GFP-vector or GFP-TIMAP^WT^ treated with thrombin (1.0 units/ml). *Left panel*, representative WB analysis. *Right panel*, quantification of densitometric data from four independent experiments: GFP-vector (■) or GFP-TIMAP^WT^ (♦) (mean ± S.D.; *, *p* < 0.01; two-way ANOVA with post hoc Bonferroni test for differences at distinct time points). *D*, glomerular ECs transduced with GFP-vector, GFP-TIMAP^WT^, or GFP-TIMAP^V64A/F66A^. pMLC2 abundance was assessed as a function of time after addition of the MLCK inhibitor ML7 (50 μm). *Left panel*, representative WB analysis. *Right panel*, densitometric analysis. Vector, ■; GFP-TIMAP^WT^, ♦; GFP-TIMAP^V64A/F66A^, ●; *n* = 4 independent experiments; *, *p* < 0.01, vector *versus* TIMAP^WT^; two-way ANOVA with post hoc Bonferroni analysis.

We next determined whether endogenous TIMAP also enhances MLC2 phosphorylation (Fig. 3, *A* and *B*). For these studies, human umbilical vein ECs (HUVECs) were used because they express endogenous TIMAP at much higher levels than glomerular ECs (data not shown). TIMAP-specific siRNA effectively reduced TIMAP protein expression in HUVECs compared with nonspecific control siRNA (Fig. 3*A*), as also observed previously (30). In ECs treated with TIMAP siRNA, pMLC2 abundance was significantly lower than in ECs treated with nonspecific siRNA (Fig. 3*A*). The pMLC2 abundance was also significantly lower in lung lysates from TIMAP-deficient compared with WT mice (Fig. 3*B*). Thus, overexpression of exogenous TIMAP and silencing or deletion of endogenous TIMAP result in enhanced and reduced MLC2 phosphorylation, respectively.

Because the effect of TIMAP on pMLC2 could be indirect, we next determined whether TIMAP alters thrombin-stimulated MLC2 phosphorylation. In GFP-vector–transduced ECs, pMLC2 increased by ∼3-fold within 5 min of thrombin addition (Fig. 3*C*). In ECs transduced with GFP-TIMAP^WT^, pMLC2 increased from a much higher baseline to levels that were essentially the same as those observed in thrombin-treated, vector-transduced cells (Fig. 3*C*). The pMLC2 levels returned to baseline 30 min after addition of thrombin in vector-transduced cells and to the much higher baseline in GFP-TIMAP^WT^-transduced cells (Fig. 3*C*). Thus, the increase in pMLC2 in TIMAP-transduced cells does not seem to be explained by greater agonist-induced MLC2 phosphorylation.

Because inhibition of myosin phosphatase increases steady-state MLC2 phosphorylation and sustained myosin-dependent contraction in smooth muscle cells (31), we wondered whether TIMAP inhibits myosin phosphatase activity in ECs. To evaluate MLC2 dephosphorylation, myosin light chain kinase (MLCK) was inhibited with ML7 (50 μm), and the level of pMLC2 remaining relative to that observed at *t* = 0 was determined as a function of time. Although pMLC2 levels remained unchanged for 60 min in vehicle-treated cells, pMLC2 fell to nearly undetectable levels in the presence of ML7, an effect that was inhibited by the PP1c inhibitor Calyculin A (Fig. S2). In vector-transduced ECs and in ECs expressing GFP-TIMAP^V64A/F66A^, pMLC2 levels fell by 75%–80% relative to baseline 60 min after ML7 addition (Fig. 3*D*). The pMLC dephosphorylation rate was significantly slower in ECs expressing GFP-TIMAP^WT^, falling only 25%–30% relative to baseline by 60 min (Fig. 3*D*). Thus, in the presence of ML7, the rate of pMLC2 dephosphorylation exceeds its rate of phosphorylation, and like Calyculin A (Fig. S2), TIMAP^WT^, but not TIMAP^V64A/F66A^, reduces pMLC2 dephosphorylation in the presence of ML7, consistent with inhibition of myosin phosphatase activity when TIMAP^WT^ is present.

### TIMAP-induced MYPT1 degradation in ECs

**Figure 4.**
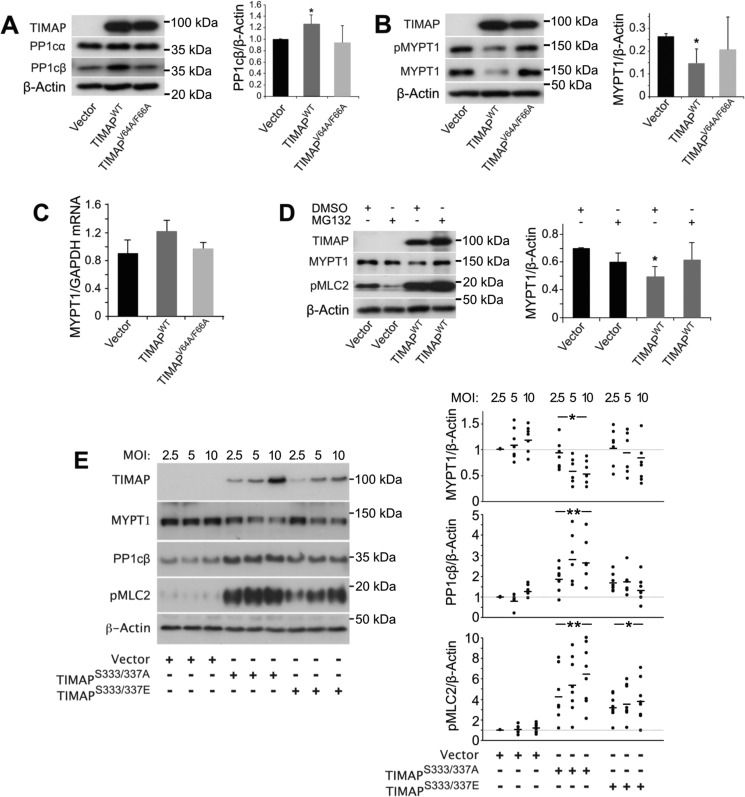
**TIMAP reduces MYPT1 abundance in ECs.** *A*, PP1cα and PP1cβ levels in ECs transduced with GFP-vector, GFP-TIMAP^WT^, or GFP-TIMAP^V64A/F66A^. *Left panel*, representative WB analysis. *Right panel*, densitometric analysis (mean ± S.D.; *n* = 5 independent experiments; *, *p* < 0.05 *versus* vector control; Student's *t* test). *B*, MYPT1 and pS696MYPT1 in ECs transduced with GFP-vector, GFP-TIMAP^WT^, or GFP-TIMAP^V64A/F66A^. *Left panel*, representative WB analysis. *Right panel*, densitometric analysis (mean ± S.D.; *n* = 5 independent experiments; *, *p* < 0.05 *versus* vector control; Student's *t* test). *C*, MYPT1 mRNA abundance determined by RT-PCR in ECs transduced with GFP-vector, GFP-TIMAP^WT^, or GFP-TIMAP^V64A/F66A^ (mean ± S.D.; *n* = 5 independent experiments). *D*, effect of MG132 (10 μm) on MYPT1 and pMLC2 abundance in glomerular ECs transduced with GFP-vector or GFP-TIMAP^WT^. *Left panel*, representative WB analysis. *Right panel*, densitometric analysis (mean ± S.D., *n* = 4 independent experiments; *, *p* < 0.05, two-way ANOVA with post hoc Bonferroni analysis). *E*, MYPT1, PP1cβ, and pMLC2 abundance in ECs transduced with 2.5, 5, or 10 m.o.i. of GFP-vector, GFP-TIMAP^S333A/S337A^, or GFP-TIMAP^S333E/S337E^. *Right panel*, representative WB analysis. *Left panel*, densitometric analysis (values for 2.5 m.o.i. of vector were set at 1.0 (*horizontal gray bar*); individual data points from six to seven independent experiments are shown; *, *p* = 0.01; **, *p* < 0.001 *versus* GFP-vector control; two-way ANOVA with post hoc Bonferroni analysis).

MYPT1/PP1cβ is the main myosin phosphatase in ECs. Because TIMAP^WT^ co-localized only partially with MLC2 (Fig. 1), we reasoned that TIMAP may regulate MLC2 phosphorylation indirectly by inhibiting MYPT1/PP1cβ activity. We therefore determined whether TIMAP overexpression alters PP1cβ or MYPT1 abundance and/or MYPT phosphorylation in ECs. Although the level of PP1cα was similar in GFP-TIMAP^WT^, GFP-TIMAP^V64A/F66A^, and GFP-vector–transduced ECs, the abundance of PP1cβ was consistently higher in ECs transduced with GFP-TIMAP^WT^ compared with ECs transduced with GFP-vector or GFP-TIMAP^V64A/F66A^ (Fig. 4*A*), in keeping with the specificity of TIMAP^WT^ for PP1cβ. By contrast, MYPT1 levels were reduced in ECs expressing GFP-TIMAP^WT^ compared with vector-transduced ECs (Fig. 4*B*). The abundance of p(Thr^696^)MYPT1 was also reduced, and this was accounted for by lower total MYPT1 levels. Overexpression of GFP-TIMAP^V64A/F66A^, which does not bind PP1cβ, did not alter the MYPT1 protein abundance. MYPT1 protein levels were also reduced in COS7 cells transfected with His-TIMAP^WT^ or GFP-TIMAP^WT^ cDNA by 31% ± 19% and 45% ± 13%, respectively (mean ± S.D., *n* = 3, *p* = 0.05 and 0.01, respectively). MYPT1 mRNA levels were not suppressed by GFP-TIMAP^WT^ (Fig. 4*C*), although the proteasome inhibitor MG132 abolished the effect of GFP-TIMAP^WT^ on MYPT1 abundance (Fig. 4*D*), consistent with enhanced proteasomal MYPT1 degradation. Nonetheless, rescuing MYPT1 from degradation with MG132 did not abolish MLC2 hyperphosphorylation in response to GFP-TIMAP^WT^ (Fig. 4*D*).

We next determined whether TIMAP phosphorylation at Ser^333^/Ser^337^, known to reduce its affinity for PP1cβ, modifies its effect on PP1cβ, MYPT1, and pMLC2 (Fig. 4*E*). Expression of GFP-TIMAP^S333A/S337A^, which cannot be phosphorylated by PKA/GSK3β and binds PP1cβ with high affinity (18), significantly raised PP1cβ and pMLC2 levels and reduced MYPT1 abundance, effects that were m.o.i.-dependent. By contrast, MYPT1 and PP1cβ levels did not differ significantly between GFP-TIMAP^S333E/S337E^ and GFP-vector–transduced EC, although TIMAP^S333E/S337E^ still increased pMLC2. Because TIMAP^S333E/S337E^ raised pMLC2 without significantly reducing MYPT1 levels, and MG132 inhibited MYPT1 degradation without abolishing TIMAP-induced MLC2 hyperphosphorylation, the findings suggest that TIMAP-induced MYPT1 degradation does not fully account for the reduced myosin phosphatase activity in ECs expressing TIMAP.

### TIMAP competes for PP1cβ with MYPT1 and blocks the PP1cβ active site

**Figure 5.**
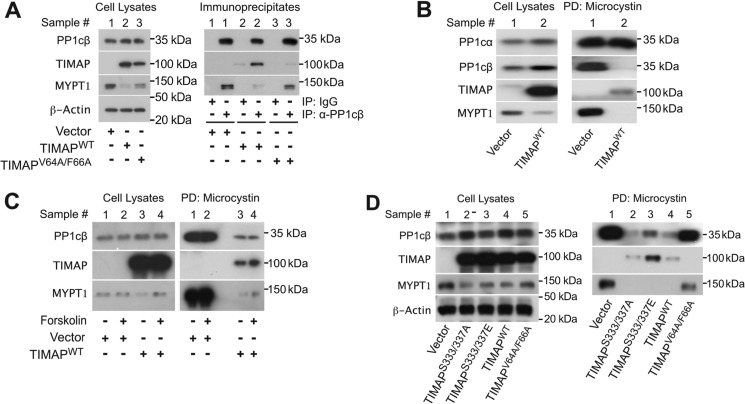
**TIMAP competes with MYPT1 for PP1cβ and blocks access to the PP1cβ active site.** *A*, PP1cβ was immunoprecipitated from lysates of glomerular ECs transduced with GFP-vector or GFP-TIMAP^WT^. Cell lysates and PP1cβ immunoprecipitates from the same samples (*Sample* #) were evaluated by WB analysis for PP1cβ, TIMAP, and MYPT1. β-Actin served as a loading control for lysates. *B*, microcystin-LR–Sepharose pulldown of PP1c from glomerular EC lysates transduced with GFP-vector or GFP-TIMAP^WT^, followed by WB analysis for PP1cα, PP1cβ, TIMAP, and MYPT1. Pulldowns (*PD*) and lysates were from the same samples. *C*, microcystin-LR–Sepharose pulldown of PP1c from glomerular EC lysates transduced with GFP-vector or GFP-TIMAP^WT^ and treated with forskolin (50 μm, 10 min) or vehicle. Cell lysates and pulldowns from the same sample were evaluated by WB analysis for PP1cβ, TIMAP, and MYPT1. *D*, microcystin-LR pulldown of PP1c from glomerular EC lysates transduced with GFP-vector, GFP-TIMAP^S333A/S337A^, GFP-TIMAP^S333E/S337E^, GFP-TIMAP^WT^, or GFP-TIMAP^V64A/F66A^. Cell lysates and pulldowns from the same sample were evaluated by WB analysis for PP1cβ, TIMAP, and MYPT1. β-Actin served as a loading control for lysates.

We next determined whether the association of PP1cβ with MYPT1 is altered by TIMAP (Fig. 5). In ECs transduced with GFP-vector, MYPT1 was readily coimmunoprecipitated with PP1cβ. By contrast, in ECs overexpressing GFP-TIMAP^WT^, the PP1cβ antibody coimmunoprecipitated PP1cβ with GFP-TIMAP^WT^ but not MYPT1 (Fig. 5*A*), indicating that GFP-TIMAP^WT^ competes for PP1cβ with MYPT1. As expected, in the presence of GFP-TIMAP^V64A/F66A^, which does not bind PP1cβ, immunoprecipitation of PP1cβ coprecipitated MYPT1 (Fig. 5*A*). We evaluated whether reduced MYPT1 protein abundance in EC expressing GFP-TIMAP^WT^ accounts for the lack of MYPT1 coimmunoprecipitation with PP1cβ in the presence of GFP-TIMAP^WT^ but found that MYPT1 was still observed in PP1cβ immunoprecipitates when MYPT1 protein expression was reduced by siRNA silencing to levels below those in the presence of GFP-TIMAP^WT^ (Fig. S4). Hence, in the presence of excess TIMAP^WT^, PP1cβ preferentially binds TIMAP over MYPT1.

To further probe the interaction of PP1cβ with TIMAP and MYPT1, we used immobilized microcystin-LR to precipitate PP1c. The cyanobacterial toxin microcystin-LR directly binds and inhibits the active site of all PP1c isoforms (32, 33). Microcystin-LR precipitated abundant PP1cα from EC lysates regardless of whether the cells were transduced with GFP-Vector or GFP-TIMAP^WT^ (Fig. 5*B*). However, although microcystin-LR precipitated PP1cβ with MYPT1 in GFP-vector–transduced ECs (Fig. 5*B*), almost no PP1cβ was pulled down by microcystin-LR in GFP-TIMAP^WT^–expressing ECs (Fig. 5*B*), even though PP1cβ was present in the EC lysates and coimmunoprecipitated GFP-TIMAP^WT^ (Fig. 5*A*). Microcystin-LR–Sepharose also failed to pull MYPT1 from ECs expressing GFP-TIMAP^WT^, consistent with depletion of PP1cβ from MYPT1 in the presence of GFP-TIMAP^WT^. Thus, microcystin-LR can interact with PP1cβ when it is associated with MYPT1, but this interaction is disrupted in the presence of GFP-TIMAP^WT^. Notably, although GFP-TIMAP^WT^ and PP1cβ associate with each other (Fig. 5*A*), microcystin-LR fails to interact with PP1cβ when it is bound to GFP-TIMAP^WT^, suggesting that the PP1cβ active site is blocked when PP1cβ is bound to TIMAP.

TIMAP is phosphorylated by PKA/GSK3β, reducing its affinity for PP1cβ (18, 19). To determine whether PKA activation results in PP1cβ dissociation from TIMAP and reassociation with MYPT1, ECs were stimulated with forskolin (Fig. 5*C*), followed by microcystin-LR pulldown. Consistent with the findings in Fig. 5*B*, substantially more PP1cβ/MYPT1 was pulled down by microcystin-LR in the absence of GFP-TIMAP^WT^ compared with the presence of GFP-TIMAP^WT^. Forskolin treatment did not mitigate the effects of TIMAP^WT^.

To further probe a potential effect of TIMAP phosphorylation, ECs were transduced with GFP-TIMAP^S333A/S337A^ or GFP-TIMAP^S333E/S337E^ to block or mimic Ser^333^/Ser^337^ phosphorylation, respectively. Compared with the GFP-vector controls, much less PP1cβ and MYPT1 were detected in microcystin-LR precipitates in cells expressing GFP-TIMAP^S333A/S337A^, GFP-TIMAP^S333E/S337E^, or GFP-TIMAP^WT^, whereas PP1cβ and MYPT1 were effectively precipitated by microcystin-LR from ECs transfected with GFP-TIMAP^V64A/F66A^, the TIMAP mutant that does not bind PP1cβ (Fig. 5*D*). Slightly more PP1cβ and TIMAP were precipitated in the presence of GFP-TIMAP^S333E/S337E^ than GFP-TIMAP^S333A/S337A^ and GFP-TIMAP^WT^ (Fig. 5*D*).

Thus, binding of PP1cβ to TIMAP strongly reduces the interaction of PP1cβ with MYPT1, indicating that TIMAP competes with MYPT1 for PP1cβ. The finding that TIMAP-associated PP1cβ is not effectively captured by immobilized microcystin-LR furthermore indicates that the PP1cβ active site is blocked when it is bound to TIMAP. Finally, TIMAP Ser^333^/Ser^337^ phosphorylation does not mitigate these effects.

## Discussion

**Figure 6.**
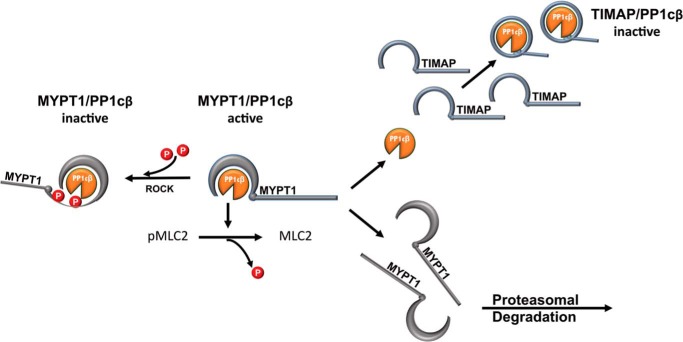
**Schematic of competition by TIMAP for PP1cβ.** The MYPT1/PP1cβ holoenzyme dephosphorylates pMLC2, inhibiting myosin function. ROCK-mediated MYPT1 phosphorylation inhibits MYPT1/PP1cβ activity. This study shows that PP1cβ preferentially binds TIMAP, resulting in proteasomal degradation of MYPT1 lacking PP1cβ. Because the active site of TIMAP-associated PP1cβ is not accessible, TIMAP inhibits PP1cβ activity.

We investigated whether TIMAP/PP1cβ acts as a functional myosin phosphatase in living ECs because TIMAP is an EC-predominant MYPT1 family member (22) that supports myosin-dependent EC processes like angiogenesis (30) and maintenance of pulmonary EC barrier integrity (34). We found that, although TIMAP partially colocalizes and directly interacts with MLC2, it inhibits MLC2 dephosphorylation in ECs by competing for PP1cβ with MYPT1, leading to MYPT1 degradation (Fig. 6). Although the interaction of PP1cβ with MYPT1 leaves the active site of PP1cβ free to bind microcystin-LR, the interaction of PP1cβ with TIMAP blocks access of microcystin-LR to the PP1cβ active site. Taken together, the data indicate that TIMAP functions as a myosin phosphatase inhibitor in ECs.

Whether TIMAP or its homolog MYPT3 can form a myosin phosphatase holoenzyme with PP1c was examined previously with mixed results. We reported that full-length TIMAP strongly inhibits *in vitro* PP1cβ phosphatase activity toward phosphorylase a, but not pMLC2 (11), although TIMAP was also reported to inhibit PP1cβ activity toward pMLC2 and pMoesin *in vitro*, an effect that was relieved by phosphorylation at Ser^333^/Ser^337^ (19). Full-length MYPT3 also inhibited PP1cγ activity toward pMLC2 (21) but had little effect on PP1cβ phosphatase activity toward pMLC2 (20). Phosphorylation of MYPT3 on Ser^340^/Ser^341^/Ser^353^ augmented pMLC2 dephosphorylation (20). Thus, unlike full-length MYPT1, which potentiates PP1cβ phosphatase activity toward pMLC2 (35, 36), full-length TIMAP and MYPT3 do not enhance but tend to inhibit PP1cβ- and PP1cγ-mediated pMLC2 dephosphorylation *in vitro*. Although this effect of TIMAP and MYPT3 is partially overcome by activating phosphorylation in the C-terminal domain, *in vitro* data so far have not shown potentiation of PP1cβ activity by TIMAP or MYPT3. Overexpression of HA-TIMAP in macrophages was without effect on baseline pMLC2 but abrogated TGF-β1-stimulated MLC2 phosphorylation (37). Whether this effect is due to TIMAP/PP1cβ-myosin phosphatase activity has not been examined. Taken together, previous evidence does not support the concept that TIMAP and PP1cβ form a myosin phosphatase holoenzyme. Instead, in agreement with the *in vitro* findings by Czikora *et al.* (19), our data suggest that TIMAP inhibits PP1cβ activity toward pMLC2.

Still, simple overexpression of PP1c targeting subunits in cells does not always reflect physiological function. For instance, ectopic expression of MYPT1 alone results in its nuclear localization and function (38, 39), and only coexpression with sufficient PP1cβ results in its usual cytoskeletal localization (38). We observed that the subcellular localization and association with myosin are similar for endogenous and overexpressed TIMAP in that overexpressed TIMAP and its mutants localize predominantly to cellular projections (18, 22), similar to the location of endogenous TIMAP (Fig. 1), that MLC2, Myosin IIA, and endogenous TIMAP all coimmunoprecipitate, and that recombinant GST-TIMAP directly binds MLC2. Finally, silencing or deletion of endogenous TIMAP in ECs and in mice, respectively, reduced pMLC2 levels, consistent with an inhibitory effect of endogenous TIMAP on PP1cβ-dependent myosin phosphatase activity. We were therefore satisfied that the effect of overexpressed TIMAP on pMLC2 reflects a physiologically relevant process, at least in ECs.

Our findings that the functional responses to TIMAP^WT^ require binding of PP1cβ to TIMAP imply that there is insufficient endogenous PP1cβ in the presence of excess TIMAP^WT^ to support myosin phosphatase activity. It seemed plausible that furnishing the ECs with more PP1cβ might unmask myosin phosphatase activity or prevent inhibition of MYPT1/PP1cβ-myosin phosphatase activity. However, TIMAP-induced MLC2 hyperphosphorylation was not altered in ECs forced to express exogenous FLAG-tagged PP1cβ along with TIMAP (40). Also, the abundance of endogenous PP1cβ consistently increased in ECs expressing TIMAP^WT^ but not TIMAP^V64A/F66A^, indicating that endogenous PP1cβ levels are regulated in response to changes in PPIP abundance, as also reported previously (41), and that increased PP1cβ expression in the presence of TIMAP^WT^ does not unmask myosin phosphatase activity.

Inhibition of myosin phosphatase activity in nonmuscle cells is frequently related to MYPT1 phosphorylation by ROCK at Thr^696^ and Thr^853^ in the MYPT1 C-terminal domain (13–15). We observed that pThr^696^-MYPT1 levels were lower in ECs expressing TIMAP^WT^ because of reduced total MYPT1 levels. Therefore, slower pMLC2 dephosphorylation in the presence of TIMAP^WT^ (Fig. 3*D*) is not due to inhibitory Thr^696^ phosphorylation of MYPT1 but may reflect, at least in part, lower total MYPT1 levels. Proteasomal degradation of MYPT1 is an established mechanism limiting myosin phosphatase activity in cells (42, 43), and MYPT1 levels fall when PP1cβ abundance is limited (41). Indeed, the proteasome inhibitor MG132 largely abrogated the TIMAP-induced reduction in MYPT1 abundance. The observations that expression of TIMAP^V64A/F66A^, which does not bind PP1cβ, did not reduce MYPT1 protein levels in ECs and that MYPT1 coprecipitated with PP1cβ in the presence of TIMAP^V64A/F66A^, but not TIMAP^WT^, imply that TIMAP^WT^, by binding PP1cβ, limits the availability of PP1cβ to MYPT1, resulting in its proteasomal degradation (Fig. 5*E*).

PP1 regulatory subunits can be purified with immobilized microcystin-LR, a cyanobacterial toxin that directly binds the PP1c active site (44, 45). Our finding that MYPT1 and PP1cβ coprecipitate with immobilized microcystin-LR is therefore not surprising, and the observation that MYPT1 is not pulled down by microcystin-LR–Sepharose in the presence of TIMAP^WT^ is consistent with competition by TIMAP^WT^ for PP1cβ. These observations are all in agreement with the view that PPIPs exist in stoichiometric excess over PP1c subunits in cells (2).

Even though TIMAP^WT^ and PP1cβ effectively coimmunoprecipitate with anti-PP1cβ (Fig. 5*A* and Fig. S3) and anti-TIMAP antibodies (11), immobilized microcystin-LR was not effective in precipitating TIMAP^WT^-associated PP1cβ (Fig. 5, *B–D*). Microcystin-LR binds the active site of all PP1c subunits with high affinity (32, 33, 46). Because PP1cβ, but not PP1cα, becomes nearly invisible to microcystin-LR in the presence of excess TIMAP^WT^, we interpret these findings to indicate that the PP1cβ active site is blocked when it is bound to TIMAP^WT^, in keeping with an inhibitory function of TIMAP on PP1cβ in ECs.

There are several well-known endogenous inhibitors of PP1c (4, 6). Among the most studied, Inhibitor-1 (I-1) and its homolog dopamine- and cAMP-regulated phosphoprotein 32 kDa (DARPP-32) are relatively small proteins that bind all PP1c isoforms with inhibitory constants in the nanomolar range. Their inhibitory activity depends on phosphorylation at specific regulatory sites (5). Inhibitor-2 (I-2) similarly binds and inhibits all three PP1c isoforms, and release of PP1c from I-2 results in its activation (7). In contrast, CPI-17 is a MYPT-specific inhibitor that binds the active site of MYPT1-bound PP1cβ in a PKC-dependent fashion. We considered the possibility that TIMAP might inhibit MYPT1/PP1cβ activity via CPI-17; however, TIMAP overexpression did not alter CPI-17 abundance or phosphorylation (40).

X-ray crystallography has shown that microcystin-LR (33), okadaic acid (47), Calyculin A (48), and tautomycin (49) all fit neatly into the PP1c active site. Similarly, the inhibitory motif I-2 occupies the PP1c active site, although there also are other contact sites between I-2 and PP1c (50). Structural modeling also indicates that pThr^38^ of CPI-17 occupies the PP1cβ active site (51) and that phosphorylated CPI-17 blocks microcystin-LR access to PP1cβ (52). This is similar to this study, where microcystin-LR does not have access to PP1cβ when it is bound to TIMAP (Fig. 5). However, although a conserved pThr residue of DARPP32 and I-1 is essential for their PP1c-inhibitory activity, DARPP32 and I-1 PP1c bind to an adjacent motif with which marine toxins do not interact (53, 54).

The idea that PP1c inhibitors can function by scavenging PP1c has been discussed (6) and would be supported by the findings that PP1c can be acutely released from its regulatory subunits. For instance, phosphorylation of the glycogen-targeting subunit (GM) by PKA on Ser in the GM KVSF motif results in PP1c release and reduced glycogen phosphatase activity (55). However, whether signaling events can be triggered through the acute exchange of PP1c subunits between inhibitory and activating PP1Ps is unclear (6). TIMAP is phosphorylated by PKA and GSK3β on Ser^337^/Ser^333^, respectively, reducing PP1cβ affinity (18, 19), and TIMAP is autodephosphorylated by its associated PP1cβ (18). We therefore examined whether TIMAP phosphorylation would result in reassociation of PP1cβ with MYPT1. However, treatment of EC with forskolin in the presence of TIMAP^WT^ or expression of Ser^333^/Ser^337^ phosphorylation-deficient or phosphomimic TIMAP mutants did not appreciably enhance reassociation of PP1cβ with MYPT1 (Fig. 5, *C* and *D*).

Although our experiments indicate that TIMAP/PP1cβ inhibits myosin phosphatase activity in ECs, it is still possible that TIMAP/PP1cβ targets other substrates; for instance, the ERM proteins moesin and merlin (17, 34, 56). In this regard, a previous study found that TIMAP^WT^ overexpression did not alter baseline ERM phosphorylation (17), similar to findings in this study (Fig. 2*B*), but overexpressed TIMAP^S331A^ enhances a TIMAP–ERM interaction with dramatic ERM dephosphorylation (17). Thus, TIMAP/PP1cβ may function as an ERM phosphatase under conditions favoring the TIMAP–ERM interaction. TIMAP also interacts directly with the 67-kDa laminin receptor (LAMR1), and LAMR1 was thought to be a TIMAP/PP1cβ substrate (57). However, LAMR1 was not a substrate for TIMAP/PP1cβ *in vitro* (11).

Our observation that microcystin-LR does not interact with TIMAP-bound PP1cβ is difficult to reconcile with a role for TIMAP/PP1cβ as a functionally active phosphatase. One would have to postulate that a small pool of TIMAP/PP1cβ associates with its substrates with an open PP1cβ active site. This is possible and might account for the small amount of PP1cβ pulled down by microcystin-LR in the presence of TIMAP (Fig. 5, *C* and *D*). It is also conceivable that TIMAP/PP1cβ substrates other than pMLC2 themselves block the PP1cβ active site, restricting access to microcystin-LR. A very transient conformational change in TIMAP could also activate or release TIMAP-associated PP1cβ. Nonetheless, the marked difference in the ability of microcystin-LR to bind MYPT1/PP1cβ *versus* TIMAP/PP1cβ strongly suggests that the active site of PP1cβ is blocked when it is bound to TIMAP (Fig. 6).

To conclude, this study indicates that endogenous and overexpressed TIMAP binds PP1cβ in ECs and blocks access to the phosphatase active site. This effect of TIMAP results in loss of MYPT1-associated PP1cβ, consequent MYPT1 degradation, and reduced myosin phosphatase activity in ECs (Fig. 5*E*). The findings that TIMAP depletion *in vivo* or silencing in cultured ECs reduces pMLC2 imply that this mechanism operates *in vivo*. Overall, our findings support the concept that distinct PPIPs can regulate the activity of PP1 holoenzymes through competition for their common catalytic subunits.

## Experimental procedures

### Reagents and antibodies

Unless otherwise specified, reagents were from Sigma-Aldrich. Polyclonal rabbit anti-pThr^18^/pSer^19^ MLC2 (catalog no. 3674), monoclonal rabbit anti-MLC2 antibody (catalog no. 8505), and monoclonal rabbit anti-MYPT1 (catalog no. 8574) were from Cell Signaling Technology (New England Biolabs, Inc.). Monoclonal mouse anti-β actin antibody (AC-15, catalog no. A5441) was from Sigma. Polyclonal rabbit anti-pMYPT1 (pThr^696^) antibody (AB S45) was from Millipore (Billerica, MA). Monoclonal mouse anti-PP1cα (E-9, sc-7482), rabbit polyclonal anti-PP1cα (FL-18, sc-443), and goat polyclonal anti-PP1cβ (C-20, sc-6106) antibodies were from Santa Cruz Biotechnology Inc. (Dallas, TX). Rabbit polyclonal anti-PP1cβ (PA1-12379) and goat anti-chicken Alexa Fluor 594 (A32759) secondary antibody were from Thermo Fisher Scientific (Rockford, IL). Control (sc-37007), hTIMAP-specific (sc-76669) and hMYPT1-specific (sc-37240) siRNAs were from Santa Cruz Biotechnology.

Polyclonal rabbit anti-TIMAP IgG was generated against the 21-mer peptide (ESSSEGKAPLIGGRTSPYSSN) representing amino acids 511–532 of hTIMAP (22). This rabbit anti-TIMAP IgG specifically recognizes bacterially expressed full-length TIMAP, ∼67-kDa endogenous TIMAP (22), knocked down by TIMAP-specific siRNA (30) and abrogated by TIMAP deletion (Fig. 3*B*). It also recognizes ectopically expressed ∼100-kDa GFP-TIMAP and TIMAP mutants not observed in vector-transduced cells (30) (Figs. 2–5). Preabsorption of rabbit anti-TIMAP IgG with the immunizing peptide fully abrogates binding on WB analysis (data not shown). Chicken anti-TIMAP IgY (57) was generated against the 19-mer peptide (NGDIRETRT DQENKDPNPN) representing amino acids 383–401 of hTIMAP. Chicken anti-TIMAP IgY immunoprecipitates produce a single rabbit anti-TIMAP IgG–reactive band (57) (Fig. S4), and chicken anti-TIMAP IgY immunofluorescence is observed only in cells expressing TIMAP (57).

### Mice

All procedures in mice were approved by the University of Alberta Animal Care and Use Committee (protocol AUP00000222). TIMAP-deficient mice were a generous gift from Prof. Conrad C. Bleul (58). The mice were backcrossed onto the C57BL/6 background for more than 10 generations. The genotype was confirmed by PCR, and TIMAP protein was absent in TIMAP-deficient mice.

### Cells

Primary human glomerular ECs and HUVECs free of mycoplasma contamination were purchased from Angio-Proteomie (Boston, MA) and maintained in EGM-2MV medium (CC3202, Lonza, Walkersville, MD). EGM-2MV consists of EBM-2 basal medium (CC3356) plus supplements (CC4147) from Lonza. Cells were grown on plates coated with Quick Coating Solution cAP-01 from Angio-Proteomie. For all experiments, ECs were used at passages 5–6. COS7 cells were maintained in DMEM containing 10% FBS.

Immunofluorescence studies were done as described previously (57). Briefly, ECs were cultured in complete medium on glass coverslips. 48 h later, they were washed, fixed with 4% paraformaldehyde, permeabilized with 0.2% Triton X-100, blocked with 10% normal goat serum, and then incubated with chicken anti-TIMAP IgY (1:5000 dilution overnight at 4 °C), followed by Alexa Fluor 594 goat anti-chicken IgG (1:4000 dilution).

### TIMAP adenovirus infection

Production of TIMAP^WT^ and TIMAP mutant constructs (18) and adenoviral vectors (30) has been described previously. For TIMAP overexpression, human glomerular ECs were used because they express relatively low levels of TIMAP at baseline (data not shown). Glomerular ECs were replated 1 day prior to infection in 35-mm culture dishes to achieve ∼70% confluent monolayers. 1 ml of EGM-2MV medium containing the appropriate adenoviral vector (2.5–40 m.o.i.) and 5 μg/ml Polybrene (Millipore, Sigma Canada) were added to each 35-mm dish. 24 h later, 1 ml of fresh EGM-2MV medium was added. Cells were harvested 48 h after adenovirus addition.

### cDNA and siRNA transfection

For transfection with His_6_- or GFP-TIMAP cDNA, COS7 cells in the logarithmic phase of replication were transfected with Lipofectamine 2000® (Life Technologies) and 4 μg of plasmid cDNA per 35-mm plate. Cells were used 48 h after transfection. His_6_- and GFP-TIMAP protein expression was verified by WB analysis.

To silence TIMAP or MYPT1 expression, ECs were plated in 35-mm culture dishes at ∼60% confluence. 24 h later, the medium was replaced with 1 ml of EBM-2 lacking antibiotics. 60 pmol of control, 60 pmol of hTIMAP-specific siRNA, or 30 and 60 pmol of control or hMYPT1-specific siRNA were added to 100 μl of Opti-MEM^TM^ (Gibco) combined with 6 μl of Lipofectamine 3000® in 100 μl of Opti-MEM^TM^, incubated for 5 min at room temperature, and then added to 1.0 ml of EBM-2 medium in each 35-mm dish. 6 h later, 1.0 ml of EBM-2 medium containing 10% FBS (no antibiotics) was added, and the cells were harvested 48 h after addition of siRNA.

### Coimmunoprecipitation

Immunoprecipitation (IP) of endogenous TIMAP was performed with chicken anti-TIMAP IgY as described previously (18, 30, 57). The ECs were washed with ice-cold PBS and harvested in cold IP lysis buffer (50 mm Tris-HCl (pH 7.5), 150 mm NaCl, 1.0% Nonidet P-40, 0.5% sodium deoxycholate, 1× cOmplete^TM^ protease inhibitor mixture, 1× PhosStop^TM^, and 100 nm Calyculin A). After homogenization on ice, insoluble material was sedimented at 17,000 × *g* for 30 min at 4 °C. The supernatant containing solubilized proteins was incubated with chicken anti-TIMAP IgY or control IgY for 1 h at 4 °C, followed by incubation with goat anti-chicken IgG beads (Aves Labs Inc., Tigard, OR) at 4 °C overnight. The beads were sedimented, washed extensively, resuspended in 2× Laemmli buffer, and denatured for 10 min at 95 °C. For each experiment, samples were cultured, processed, and evaluated concurrently with the same antibody preparation.

### WB analysis and quantification

EC monolayers were washed once with ice-cold PBS and immediately lysed in 150 μl of 2× Laemmli sample buffer and heat-denatured for 10 min at 95 °C. Lung tissue lysates were heat-denatured at 95 °C for 10 min in Laemmli sample buffer. Proteins were separated by SDS-PAGE and transferred to PVDF membranes. Total and phosphorylated proteins were detected by probing with appropriate primary and HRP-conjugated secondary antibodies, followed by ECL (GE Amersham Biosciences, Baie d'Urfe, QC, Canada). The membranes were exposed to X-ray film (Fuji Medical X-Ray Film Super Rx, Fujifilm). Band density was evaluated using ImageJ (National Institutes of Health). For TIMAP WB analysis, transfer to PVDF membranes was done at 4 °C and 40 V for 16–20 h, blocking (Sigma, WO138) for 16–20 h, incubation with rabbit anti-TIMAP IgG (1:400) in the same blocking solution at 4 °C for 16–20 h, followed by HRP-conjugated goat anti-rabbit IgG (1:5000) for 90 min at room temperature.

### Quantitative RT-PCR

Total RNA from ECs in 60-mm plates was prepared with the RNeasy Mini Kit (74104, Qiagen). 1.0 μg of total RNA was reverse-transcribed using qScript cDNA SuperMix (VWR) in a 20-μl reaction volume. Samples lacking reverse transcriptase controlled for amplification from genomic DNA. The cDNA samples were diluted 10-fold, and 1 μl was subjected to real-time qPCR using RT^2^-SYBR Green qPCR Mastermix (Qiagen, catalog no. 33050) and RT^2^-qPCR primer for human MYPT1 (PPP1R16A) (Qiagen, PPH18472C) with the 7500 Real Time PCR System (AB Applied Biosystems). The primer pair for human hypoxanthine-guanine phosphoribosyltransferase (Qiagen, QT00059066) controlled for loading. The quantitative real-time PCR cycles were as follows: 95 °C for 10 min, followed by 40 cycles of 95 °C for 15 s and 60 °C for 1 min. The ΔΔCT method was used to calculate relative MYPT1 expression.

### Microcystin-LR–Sepharose affinity coprecipitation

Microcystin-LR–Sepharose was prepared as described by Moorhead *et al.* (45) and washed twice with 500 μl of buffer A (50 mm Tris-HCl (pH 7.4), 0.1 mm EDTA, 0.5 mm MnCl_2_, and 0.2% β-mercaptoethanol) prior to use. Cells in 100-mm dishes were lysed in 0.9 ml of lysis buffer (0.5% NP40, 25 mm Tris-HCl (pH 7.5), 137 mm NaCl, 1 mm EDTA, 5% glycerol, 1 mm DTT, and 1× cOmplete^TM^ protease inhibitor mixture) for 15 min at 4 °C, washed once with 10 ml of ice-cold PBS, followed by centrifugation at 14,000 × *g* at 4 °C for 20 min. 100 μl of the soluble supernatant was reserved, and 750 μl was incubated with 50 μl of microcystin-LR–Sepharose overnight at 4 °C with end-over-end rotation. The beads were then washed three times with buffer A containing 150 mm NaCl and 0.1% Tween 20. Proteins were eluted from the beads with 70 μl of 2× Laemmli sample buffer for subsequent SDS-PAGE electrophoresis and WB analysis. For each experiment, all samples were evaluated concurrently with the same microcystin-LR–Sepharose preparation.

### Statistics

All experiments were repeated three or more times as indicated. Data are presented as mean ± S.E. or mean ± S.D., as appropriate. For comparison of two groups, two-tailed Student's *t* test was used. Comparison of more than two groups was done by two-way ANOVA followed by post hoc Bonferroni test. *p* < 0.05 was considered significant.

## Author contributions

X. W., M. O., C. F. B. H., and B. J. B. conceptualization; X. W., M. O., L. L., and S. A. data curation; X. W., M. O., L. L., P. P., S. A., and B. J. B. investigation; X. W., M. O., L. L., P. P., S. A., and B. J. B. methodology; M. O. and B. J. B. formal analysis; L. L., C. F. B. H., and B. J. B. supervision; C. F. B. H. and B. J. B. resources; C. F. B. H. and B. J. B. funding acquisition; C. F. B. H. and B. J. B. writing-review and editing; B. J. B. validation; B. J. B. writing-original draft; B. J. B. project administration.

## Supplementary Material

Supporting Information

## Footnotes

6 The abbreviations used are:

PPIP: PP1c-interacting protein
MYPT: myosin phosphatase target
MLC: myosin light chain
ROCK: Rho-associated kinase
ERM: Ezrin Radixin Moesin
EC: endothelial cell
HUVEC: human umbilical vein endothelial cell
MLCK: myosin light chain kinase
WB: Western blot
cDNA: complementary DNA
IP: immunoprecipitation
qPCR: quantitative PCR
ANOVA: analysis of variance
TIMAP: transforming growth factor-β inhibited membrane associated protein
hTIMAP: human TIMAP.
