# Supplementary material for: TIMAP inhibits endothelial myosin light chain phosphatase by competing with MYPT1 for the catalytic protein phosphatase 1 subunit PP1cβ
Source: J Biol Chem. 2019 Jul 17;294(36):13280–91. doi: 10.1074/jbc.RA118.006075 (PMC6737228; doi:10.1074/jbc.RA118.006075)
Supplement: Supporting Information [file supp_294_36_13280__index.html]

TIMAP inhibits endothelial myosin light chain phosphatase by competing with MYPT1 for the catalytic protein phosphatase 1 subunit PP1cβ — TIMAP competes for and inhibits PP1cβ — TIMAP inhibits endothelial myosin light chain phosphatase by competing with MYPT1 for the catalytic protein phosphatase 1 subunit PP1cβ — TIMAP competes for and inhibits PP1cβ — Supporting Information 

# TIMAP inhibits endothelial myosin light chain phosphatase by competing with MYPT1 for the catalytic protein phosphatase 1 subunit PP1cβ

## Supporting Information

- Supporting Information (to be published online) - Supporting Data
