# Supplementary material for: TIMAP inhibits endothelial myosin light chain phosphatase by competing with MYPT1 for the catalytic protein phosphatase 1 subunit PP1cβ
Source: J Biol Chem. 2019 Jul 17;294(36):13280–91. doi: 10.1074/jbc.RA118.006075 (PMC6737228; doi:10.1074/jbc.RA118.006075)
Supplement: Supporting Information [file supp_RA118.006075_140985_1_supp_362619_pttstx.pdf]

Supporting Figure S1

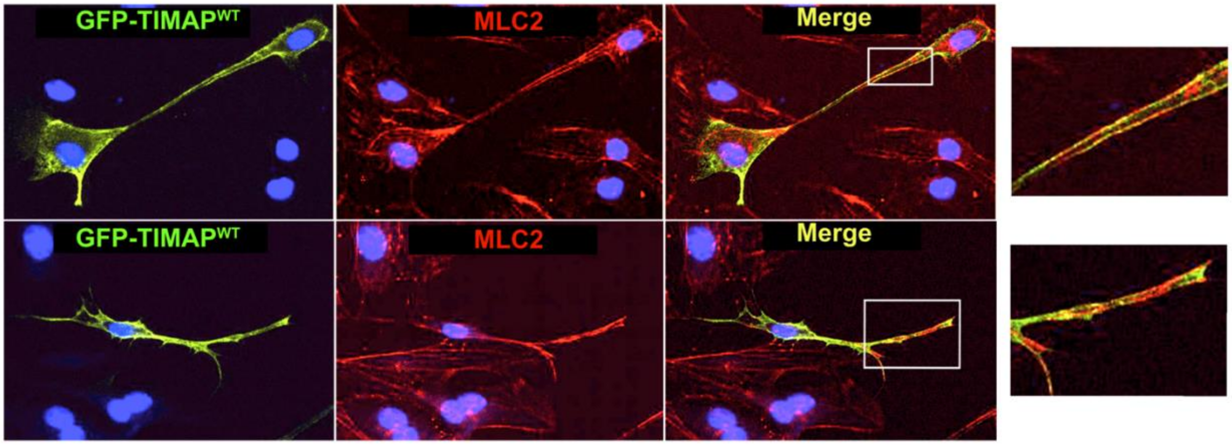

Figure S1: *Co-localization of overexpressed GFP-TIMAP^{WT} with MLC2 in glomerular EC.* Glomerular EC were plated on collagen-I coated glass coverslips, in 35-millimeter plates. Cells in the logarithmic phase of growth were transiently transfected with GFP-TIMAP cDNA using 4µg cDNA and Lipofectamine Lipofectamine 2000® (Life Technologies) . 48 hours after transfection, cells were fixed with 4% paraformaldehyde for 20 min, permeabilized with 0.02 % Triton X-100, blocked with 10% goat serum in PBS for 1 hour, followed by incubation at 4°C overnight with rabbit anti-MLC2 (1:500; . The cells were then washed extensively with 5% bovine serum albumin in PBS. Native GFP (green) and MLC2 (visualized with Alexa Fluor 594 goat anti-rabbit IgG red), respectively. Yellow fluorescence in the merged images indicate co-localization. The areas in the white boxes were digitally magnified.

Supporting Figure S2

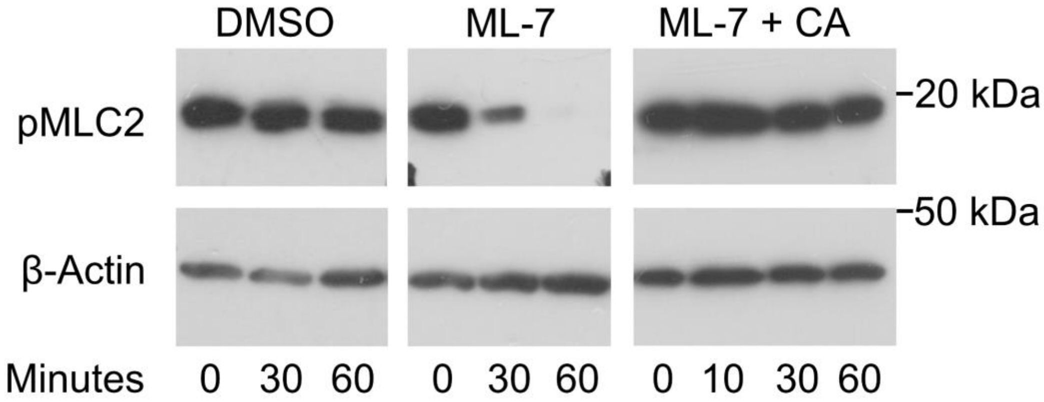

Figure S2: *Rapid pMLC2 dephosphorylation in ML-7-treated glomerular EC is blocked by the PP1c active site inhibitor Calyculin A.* Confluent glomerular EC were treated with vehicle (DMSO), the myosin light chain kinase (MLCK) inhibitor ML-7, or both ML-7 and Calyculin A for 60 min. The abundance of pMLC2 was assessed as a function of time after addition of ML7.

Supporting Figure S3

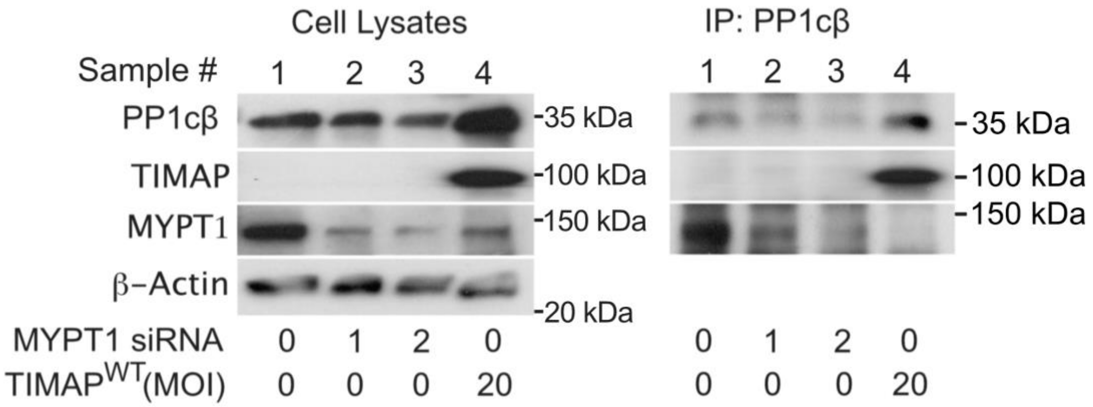

Figure S3: *MYPT1 silencing reduces but does not abolish MYPT1 co-precipitation with PP1cβ.* Glomerular EC at ~ 70% confluence in 35 mm plates were transfected with 60 pmol control siRNA (Sample #1), 30 pmol (Sample #2) or 60 pmol (Sample #3) MYPT1-specific siRNA or transduced with 20 MOI Ad-GFP-TIMAP^{WT} (Sample #4). Lysates from each sample were subjected to immunoprecipitation with goat anti-PP1cβ IgG. Total lysate and immunoprecipitates from each sample were subjected to WB analysis for PP1cβ, TIMAP and MYPT1. β-actin served as the loading control for lysates.

Supporting Figure S4

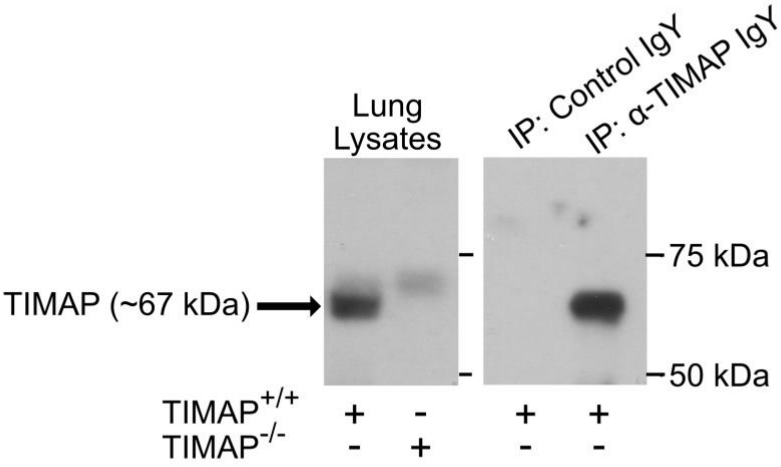

Figure S4: *TIMAP Antibody Specificity.* Left Panel: Lung lysates prepared from one wild-type (TIMAP$^{+/+}$) and one TIMAP deficient (TIMAP$^{-/-}$) mouse were subjected to WB analysis (Lung Lysates) using polyclonal rabbit anti-TIMAP IgG. Right Panel: Lung lysate from one TIMAP$^{+/+}$ mouse was subjected to immunoprecipitation (IP) with control chicken IgY or chicken anti-TIMAP IgY followed by WB analysis of the precipitates with polyclonal rabbit anti-TIMAP IgG. Endogenous TIMAP (~67 kDa) was detected in TIMAP$^{+/+}$, but not TIMAP$^{-/-}$ lung lysates. Chicken anti-TIMAP IgY immunoprecipitated the same ~67kDa rabbit anti-TIMAP reactive protein from lung lysate of the TIMAP$^{+/+}$ mouse. The ~70 kDa band detected by rabbit anti-TIMAP IgG in lung lysates is nonspecific because it is also observed in lung lysates of the TIMAP$^{-/-}$ mouse and is not immunoprecipitated with chicken anti-TIMAP IgY.
